# Towards a more just post-coal future: Engaging local communities in post-mining landscape restoration through participatory mapping methods

**DOI:** 10.1007/s13280-026-02386-y

**Published:** 2026-04-04

**Authors:** Lucia Brisudová, Bohumil Frantál

**Affiliations:** Department of Geography, Faculty of Science, Palacky University Olomouc, Olomouc, Czech Republic

**Keywords:** Just transition, Landscape restoration, Participatory mapping, Post-mining land-use, Procedural justice

## Abstract

Although energy transition policies are expected to adhere to the principles of procedural, distributive, recognition, and restorative justice, there remains a significant gap in both practice and research regarding public participation in post-mining land-use planning. Using a case study of a closing open-cast mine in Most district, Czech Republic, drawing on responses from 101 local residents, we demonstrate the efficacy of combining a participatory mapping approach with statistical and spatial analysis to engage local stakeholders in the research. It also highlights the importance of integrating community perspectives and interests into landscape assessment and post-mining land-use planning. Adoption of the approaches and methods presented in this study can enable post-mining regeneration planners to understand better the sense of place, landscape values, and needs of local communities. This, in turn, can foster place-making on post-mining sites, help prevent land-use conflicts, and contribute to more just post-coal development.

## Introduction

Post-mining landscape restoration and recycling is a global challenge that involves many dilemmas, often provoking land-use conflicts and reproducing environmental injustices (Pasqualetti and Frantál 2022; Brock 2023; Frantál et al. 2024; Klusáček et al. 2025). Conflicts typically arise due to differing stakeholder preferences for post-mining land uses. Addressing the needs and preferences of local communities is crucial to upholding the principles of the just transition, which comprise four aspects: procedural, distributive, recognition, and restorative justice (Abram et al. 2022). The restorative aspect emphasises the redressing of past harms, which is particularly relevant for coal-mining areas and their communities that have been constituted in the past as “energy sacrifice zones” (Bell 2016; Klusáček et al. 2025). As pointed out by Beckett and Keeling (2019), post-mining restoration projects present a unique opportunity to negotiate and articulate morals, values, histories, experiences, harms, and injustices associated with coal-mining landscapes, and they can represent a *“creative process of landscape and community healing”*.

Although energy transition policies are expected to adhere to the principles of environmental justice, there remains a significant gap in both practice and research regarding public engagement in post-mining land-use planning. To address this research gap and contribute to more inclusive post-mining land-use planning, this study employs a participatory mapping method to examine the local community’s perceptions and preferences regarding the future use of the post-mining landscape in the town of Horní Jiřetín, in the Most district, Czech Republic. Applied participatory mapping offers a spatially explicit approach to capturing local knowledge, preferences, and values, enabling communities to visualise and articulate their visions for landscape regeneration (Garcia-Martin et al. 2017). By engaging local residents in mapping their preferred future land uses, this approach not only fills the identified research gap but also provides insights into whether participatory spatial methods can effectively support just transition principles in post-mining contexts. The findings contribute to understanding how community-centred approaches might enhance the legitimacy and social acceptance of post-mining restoration projects while addressing historical environmental injustices.

The objectives of the study are twofold. The methodological objective is to demonstrate the feasibility of using participatory mapping as a research method in the context of planning and decision-making for the restoration of the landscape after coal mining and to test its strengths, advantages, and limitations. The empirical objective is to map and analyse differences in residents’ preferences for post-mining land use, identifying spatial consensus patterns and potential conflicts in community landscape restoration visions. This is achieved through face-to-face participatory mapping with grid-based preference recording and statistical analysis of spatial agreement patterns. Specific research questions that have driven our empirical study were defined as follows:

1. What are the spatial patterns of residents’ preferences for specific post-mining land use scenarios in the study area?
2. What is the level of local support for leaving post-mining areas to natural succession versus managed reclamation of the landscape for socioeconomic and cultural needs?
3. Do residents’ preferences for specific post-mining land uses differ according to their sociodemographic characteristics?

## Theoretical background

### Dilemmas of post-mining restoration

A fundamental dilemma associated with the post-mining transformation stems from differing values, knowledge, and competing ideas about future land use from the point of view of different stakeholders (Martinát et al. 2016; Kivinen et al. 2018; Poudyal et al. 2024). In recent years, arguably the most discussed issue in the context of post-mining transformation is the long-term prioritisation of technically and financially demanding reclamation activities at the expense of natural succession (Prach and Hobbs 2008; Frantál et al. 2024). Natural or ecological succession is considered the process of spontaneous, self-driven recovery of ecosystems on disturbed land without human intervention (Walker et al. 2007; Pešout et al. 2022). Spontaneous or managed succession can create a “new wilderness” that becomes not only a refuge for endangered plant and animal species but also a place with significant aesthetic, recreational, and tourist potential (Wiegleb and Felinks 2001; Hendrychová et al. 2020; Braun Kohlová et al. 2021). However, the possibility of leaving the post-mining landscape to spontaneous natural succession encounters legislative restrictions, traditional thinking of experts and politicians, and protests from local communities with different preferences for the future of “their” landscape (Krzysztofik et al. 2020; Klusáček et al. 2025). Although natural succession is relatively cheap, it is a long-term process without direct economic benefits (Bradshaw 2000).

Traditional reclamations carried out since the 1950s (see e.g. Tischew and Kirmer 2007; Krümmelbein et al. 2012) in the form of reforestation (sometimes in the form of pine or spruce monocultures), flooding and creating lakes, or cultivation of land for agricultural production are facing local impacts of climate change (i.e. drought and greater water evaporation, lack of water sources, forest fires, landslides, etc.), and some turn out to be environmentally and economically unsustainable (Bell et al. 2017; Pešout et al. 2022). Natural hazards and potential risks associated with climate change have not always been considered when setting reclamation goals. Post-mining lakes, which have been highlighted in recent years, can serve as spectacular examples of landscape renewal for recreation (de Waal and de Wit 2012; Deshaies 2018; Kantor-Pietraga et al. 2023). However, these lakes may also face water resource constraints or poor water quality, and they can pose future risks to the environment and local communities (Soni et al. 2014; Lund and Blanchette 2023).

The post-mining areas also have significant potential to generate renewable energy (Alves Dias et al. 2018; Pasqualetti and Smardon 2025). It is not only about solar and wind parks but also about biomass production, geothermal energy, and opportunities in hydropower and pumped-hydroelectric storage. Especially in Germany, extensive areas after surface coal mining have been used for renewable energy production (Topaloglou et al. 2024). The use of post-mining areas for the installation of solar panels and wind turbines has resulted in some areas in Lower Lusatia almost entirely disappearing as legacies of mining, which can be considered a “trivialisation of the landscape” (Deshaies 2018). Regions or localities that have been “sacrificed” for coal mining and energy production in the past can easily become new “green sacrifice zones” (areas devoted to projects supporting global energy transition and decarbonisation, such as renewable energy developments, lithium extraction, or green technology manufacturing, that paradoxically inflict environmental or social harm on local communities, see e.g. Scott and Smith 2017) and subjects of so-called green extractivism (Brock et al. 2021; Archer and Calvão 2024). In this regard, the interests and preferences of both regional governments and local communities must be considered, and the principles of just transition must be strictly applied in practice.

Another dilemma associated with post-mining transformation concerns the extent to which post-mining remnants should be preserved as cultural heritage and used for tourism development (Kaźmierczak et al. 2019) or eliminated to rebrand local and regional identity (Frantál et al. 2024). In many regions, the remnants of mining have been integrated into regenerated recreational lakelands as reminders of the industrial past, landmarks, and tourist attractions (Deshaies 2018; Lamparska 2019). The post-mining redevelopment options should be assessed in relation to local natural and climatic conditions, the extent of the affected area, the proximity of human settlements, the natural, cultural, and economic potential, and related risks (Marot and Harfst 2021; Kivinen et al. 2018).

### Public engagement in post-mining land use planning

Post-mining landscape regeneration presents complex challenges that require inclusive planning approaches that incorporate diverse perspectives, particularly those of local communities who directly experience mining impacts and reclamation outcomes. Traditional top-down approaches to post-mining land-use planning have often privileged technical and economic considerations while marginalising local knowledge and preferences. Engaging local communities represents a crucial strategy for identifying solutions that balance economic and environmental imperatives with social acceptability. This is especially important in contexts where communities maintain nostalgic ties to mining locations and where regional identity and place attachment remain strongly linked to the industrial past (Svobodová 2019). This recognition has driven the gradual adoption of participatory mapping techniques to capture spatial perceptions, values, and preferences in post-mining contexts. Participatory mapping methods were effectively applied across diverse planning scenarios, including strategic urban planning (Brisudová and Klapka 2023), urban safety (Šerý et al. 2023), urban thermal comfort (Květoňová et al. 2024), as well as in perceptions of health risks (Maestripieri and Saqalli 2016) and natural hazards (Ahmed et al. 2019).

Despite their proven effectiveness across various planning contexts, participatory mapping methods have yet to realise their full potential as established tools in post-mining regeneration (Everingham et al. 2018; Kozłowska-Woszczycka and Pactwa 2024).

The Perception-Based Regional Mapping (PBRM) methodology offers a structured, cost-effective approach to capturing local spatial knowledge through mental maps, integrating qualitative perceptions with quantitative spatial data through participatory mapping interviews (Saqalli et al. 2009). Maestripieri and Saqalli (2016) demonstrated PBRM’s versatility by applying it to identify environmental health hazards across three sites, including a mineral raw materials mining area, thereby enabling communities to map their risk perceptions spatially.

Along with the rapid evolution of geospatial technologies, including GIS, in recent decades, a new suite of participation tools known as Participatory GIS (PGIS) or Public Participation GIS (PPGIS) has emerged (Sieber 2006). With the primary objective of “*making GIS and other spatial decision-making tools available and accessible to all those with a stake in official decisions*” (Schroeder 1996 as cited in Sieber 2006, p. 492), PPGIS approaches have emerged as powerful participatory tools. These tools, represented by various online mapping interfaces, have rapidly gained popularity across diverse studies seeking to incorporate stakeholder opinions, including those of local citizens, into decision-making processes. Given this broader trend towards participatory spatial planning, PPGIS has emerged as a prominent participatory mapping approach in post-mining contexts, as illustrated by the following examples. Recent studies have applied PPGIS to post-mining landscape planning, employing various technological platforms and methodological approaches. These applications range from comprehensive community engagement platforms (e.g. ArcGIS Survey123; Kozłowska-Woszczycka and Pactwa 2024) to specialised stakeholder group perception-mapping tools (e.g. Harava online map-based survey; Kivinen et al. 2018). Some PPGIS implementations capture both current conditions and historical experiences of mining impacts (Onencan et al. 2018). In contrast, others focus primarily on present-day landscape perceptions (Santé et al. 2020), and still others concentrate specifically on validating critical local expert knowledge about mining landscapes (Ang et al. 2021).

Additional studies focusing on mining and post-mining landscapes employ participatory mapping tools but use more specific terminology. For instance, Svobodová et al. (2021) utilised what they termed “participatory value mapping”. While the authors present this method as a form of PPGIS approach, participants used printed topographic maps and coloured pencils to identify and map places with different values in their mining communities, thereby classifying the method as sketch mapping (Denwood et al. 2022; Brisudová 2024). Another example is the study by Pietta and Tononi (2021), who employed a collaborative methodology combining Participatory Action Research (PAR) (Kindon et al. 2007) with Cultural Ecosystem Services (CES) mapping to create a participatory process that engaged the local community in identifying and mapping places of cultural value. This approach also utilised printed maps and aerial photographs, suggesting it could similarly be classified as sketch mapping.

Regardless of the participatory mapping approach employed, the benefits of these methodologies are undeniable. As Maestripieri and Saqalli (2016, p. 732) note, “*Seeing the place like people see it strongly supports the endowment of local perspectives and the understanding of people’s behaviours*”—a principle particularly relevant in coal mining contexts where complex historical and cultural factors shape landscape perceptions. As Svobodová and Everingham (2026, p. 798) emphasize in their recently published theoretical essay, to fully realise the potential of PPGIS in mining contexts, it should have been ideally integrated across the entire mine life cycle, not as a one-off engagement tool, but as a continuous, adaptive process.

## Materials and methods

### Regional context and case study area

**Fig. 1:**
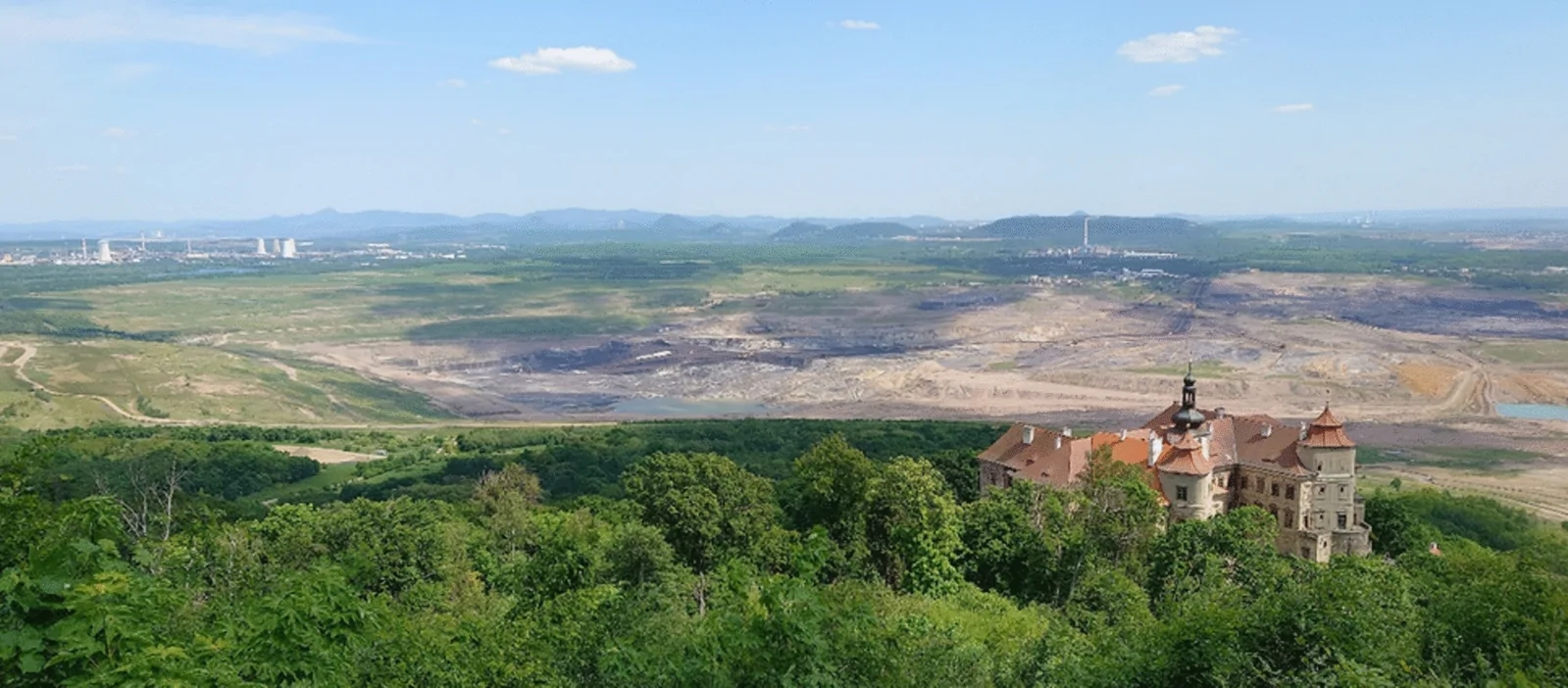
View of the Czechoslovak Army Mine from the hill above Jezeří Castle. Photograph: Authors

The Czechoslovak Army Mine (hereinafter CSA mine) is located in the Most district in the northwest of the Czech Republic on the border with Germany (Frantál et al. 2022). The total area of the open-cast mine was approximately 45 sq. km (see Fig. 1). In history, the Komorany Lake was located on the territory of the quarry, and it was drained in the middle of the nineteenth century, which enabled the agricultural use of the landscape and subsequently the development of coal mining (Říha et al. 2005). The town of Horní Jiřetín with a population of 2200 is located right on the border of the quarry. The town has been threatened by the spatial expansion of mining for several decades, and its development has been largely constrained (Černoch et al. 2019). Its future development is now closely linked to plans for the restoration and new use of the post-mining landscape.

**Fig. 2:**
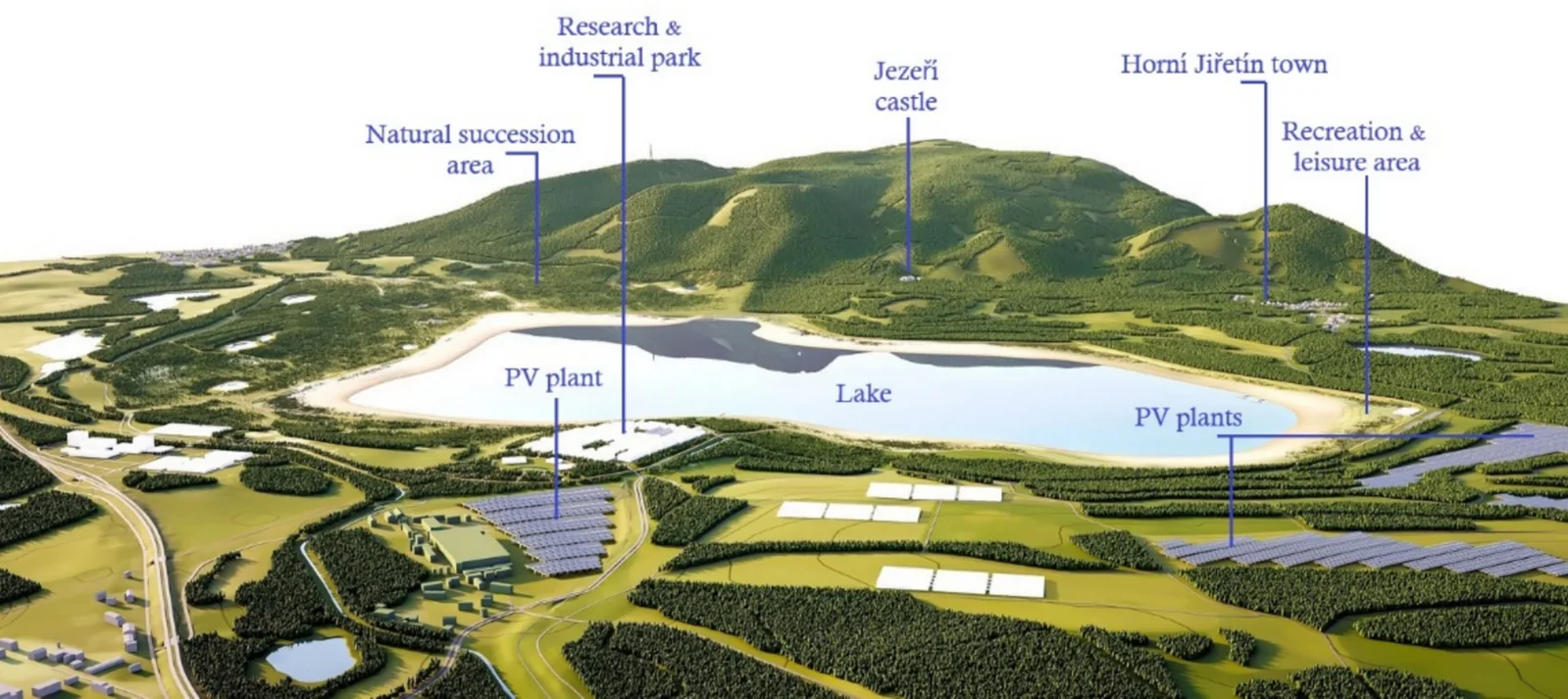
Visualisation of the future form of the area according to the Green Mine project. *Source*: Sev.en Inntech (www.greenmine.cz), adapted by the authors

In 2024, the mining company announced the suspension of quarry operations and the dismissal of most of its employees. Various scenarios for landscape restoration and new development have recently been discussed and have become the subject of controversy among experts, politicians, environmentalists, and local communities. Recently a strategic project called “Green Mine” which has been supported by the Czech Government and regional authorities (to be funded from the EU Operational Programme Just Transition) has been initiated to restore the landscape after mining and to help socioeconomically develop the area and a wider region (see Fig. 2). The key points of contention are the extent of flooding and the creation of a new lake, the size of the planned nature reserve (on which a spontaneous natural succession should create a new wilderness), and the development of new forms of energy production in the form of photovoltaic plants, “green” hydrogen production, and pumped storage power plant (see Klusáček et al. 2025). The CSA mine is intended to serve as a model for the post-coal transformation of other mines in the region; therefore, the stakes for its restoration are high.

### Research methods and data processing

As outlined in the introduction, this study employed a participatory mapping technique, specifically retrospective sketch mental mapping, to capture residents’ spatial perceptions of landscape values and preferences for post-mining landscape regeneration. Mental mapping refers to the graphic representation of an individual’s perception and subsequent cognitive conceptualisation of geographical space (Drbohlav 1991), reflecting significantly subjective and socioculturally conditioned attitudes, values, opinions, and beliefs (Golledge and Stimson 1997). Following the classification of mental mapping methods by Denwood et al. (2022), we implemented sketch mapping—a non-digital technique in which spatial data are collected directly on physical base maps. The retrospective character of our approach refers to the elicitation of spatial information from participants’ memory-stored knowledge, in contrast to in situ mapping methods that capture immediate perceptions (for further methodological distinctions, see Brisudová 2024). To operationalise this methodological approach, we developed a standardised mapping protocol with carefully designed materials and procedures that were part of the survey.

Each respondent was provided with a base map of the study area, printed in A3 format, and enclosed in a transparent, protective punched pocket. The paper map consisted of a cartographic base derived from the national map series of the Czech Republic (1:25 000 scale) and the official municipal boundaries of Horní Jiřetín. To facilitate systematic spatial analysis, the study area was subdivided into a grid network of 580 squares, each measuring 350 m on a side. This specific grid resolution was selected through careful consideration of two competing factors: the need to maintain sufficient spatial granularity for distinguishing fundamental landscape characteristics (particularly the differentiation between natural and built environments), while simultaneously preserving the visual clarity and interpretability of the underlying topographic features. Each grid cell was assigned a unique numerical identifier (1–580) to enable precise spatial referencing during subsequent analysis. This methodological design enabled rigorous spatial analysis while preserving the map’s readability for participants.

To facilitate the mapping exercise, participants were provided with six distinct waterproof markers in different colours. This combination of transparent pockets and coloured markers enabled precise mapping while preserving the visibility of the underlying base map throughout the exercise.

The participatory mapping protocol consisted of two sequential spatial tasks. The first task focused on identifying landscapes of significant value, where participants were asked to delineate areas they considered valuable and categorise them according to five predefined value types: (1) natural, (2) cultural–historical/spiritual, (3) residential, (4) recreational, and (5) productive values. The second task addressed preferences for the post-mining land-use of the mine area. Participants were instructed to designate areas for five potential land-use types: (1) natural succession, (2) planned greenery, (3) housing development, (4) recreation and tourism, and (5) renewable energy/industrial development.

When selecting categories of landscape values and landscape functions for our study, we based our selection on typologies used in surveys of land-use planning and natural resource management (e.g. Brown 2004; Willemen et al. 2008). The final selection was based primarily on the specific case study area’s conditions (reflecting ongoing disputes about possible future uses of the landscape) and on our assumptions regarding the practical implementation of the research (plotting areas in different colours on the map and the limits of clarity for processing and analysis).

**Fig. 3:**
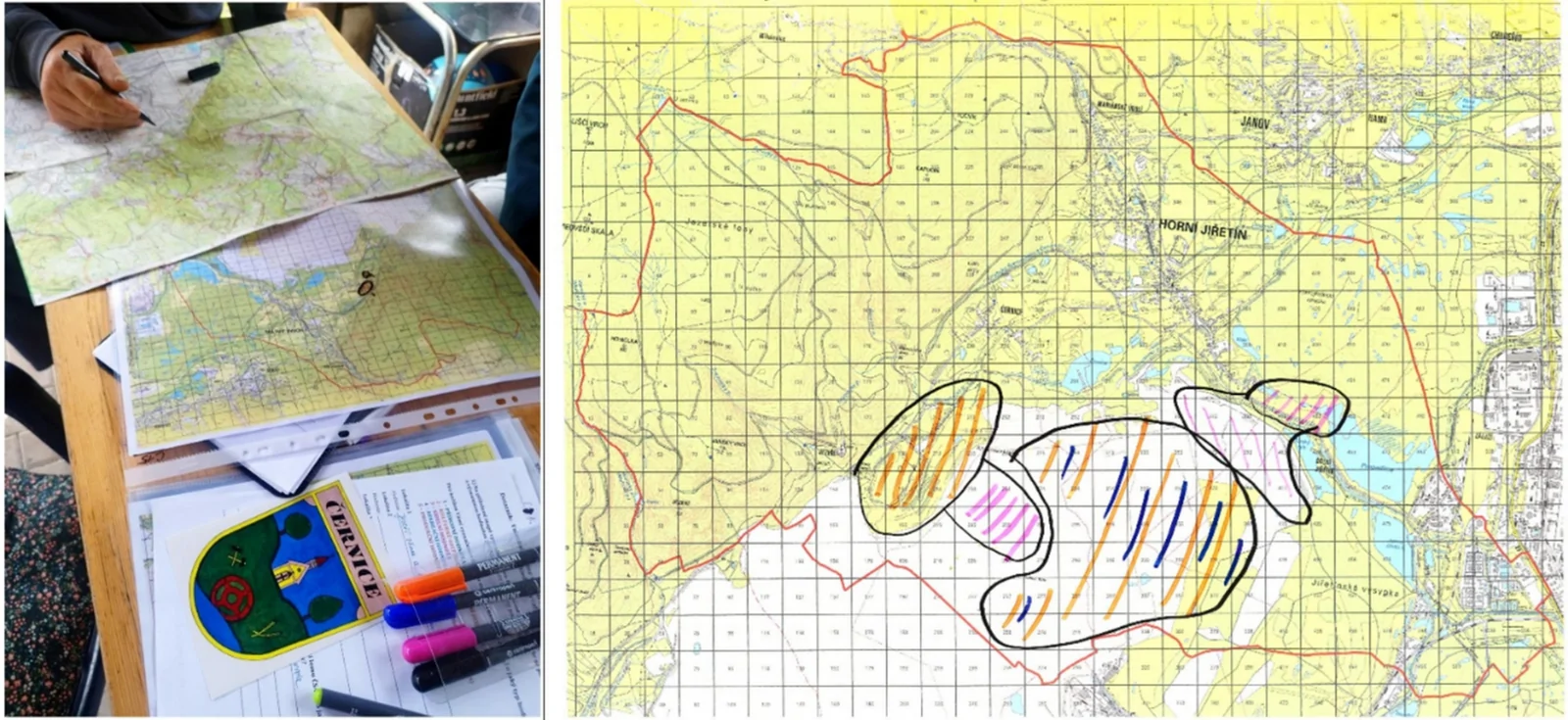
An example of participatory mapping output illustrating an individual respondent’s vision for the preferred post-mining land use (number of squares in square grid = 580). Photograph: Authors

As noted above, participants were provided with different coloured markers, each corresponding to a specific land-use preference category (e.g. green for natural succession, blue for recreation and tourism). They were asked to indicate their preferred future land uses by marking areas on the transparent overlay using these colour-coded markers (see an example in Fig. 3). The mapping protocol allowed participants complete freedom in terms of the number and extent of areas they could delineate, ensuring a comprehensive capture of their spatial preferences and perceptions.

The subsequent digitisation process involved a systematic transcription of the spatial data from the transparent overlays into a digital format represented by the numerical database. Specifically, each coloured marking on the transparent overlays was transcribed into an Excel spreadsheet by recording the marked grid cell numbers (1–580) and their associated land-use categories as indicated by the colour coding. This method transformed qualitative spatial preferences into a structured dataset suitable for subsequent analysis.

**Fig. 4:**
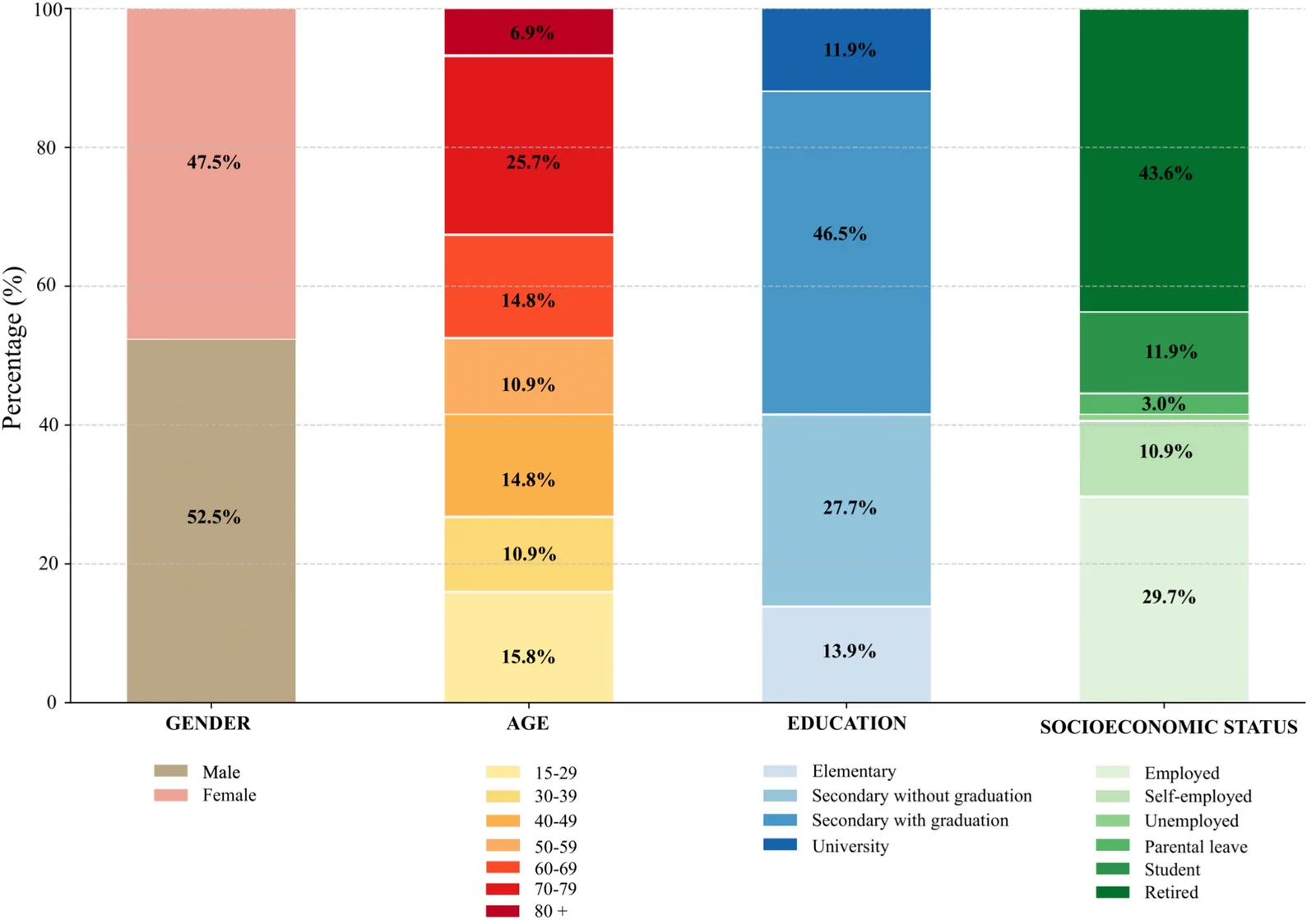
Basic sociodemographic characteristics of the research sample (*N* = 101). *Source*: authors’ survey

The survey was conducted over three days in June 2024 in the town of Horní Jiřetín. The data collection team comprised three trained research assistants and two principal investigators (authors of the paper). The survey, consisting of a questionnaire with a few closed and open-ended questions and a participatory mapping exercise described above, was implemented through on-site interviews (in people’s households or directly on the street, for example, at a bus stop or in a restaurant garden). The sampling method was a combination of convenience sampling (people’s availability at a certain time, willingness to participate in research) and quota sampling (the aim was to have a representation of all age and education categories, residents from different parts of the town, with different lengths of residence in the area). The study comprised 101 residents aged 15 and above, representing 5.3% of the town’s population aged 15 years and older. The basic characteristics of the sample are shown in Fig. 4. We acknowledge that it was not a random sampling and some population groups are slightly overrepresented (particularly older adults aged 60 years and older). However, the primary objective of this study was not to generalize results to the entire population but to test and validate the methods and tools employed and explore specific spatial and social differences in preferences for post-mining land use.

### Spatial data analysis

Spatial analysis encompassed the entire Horní Jiřetín cadastre, including surrounding forests, lakes, and other natural features, thereby ensuring participants could express preferences without artificial spatial barriers. Two complementary analytical approaches were employed to examine both preference patterns and consensus levels.

**Fig. 5:**
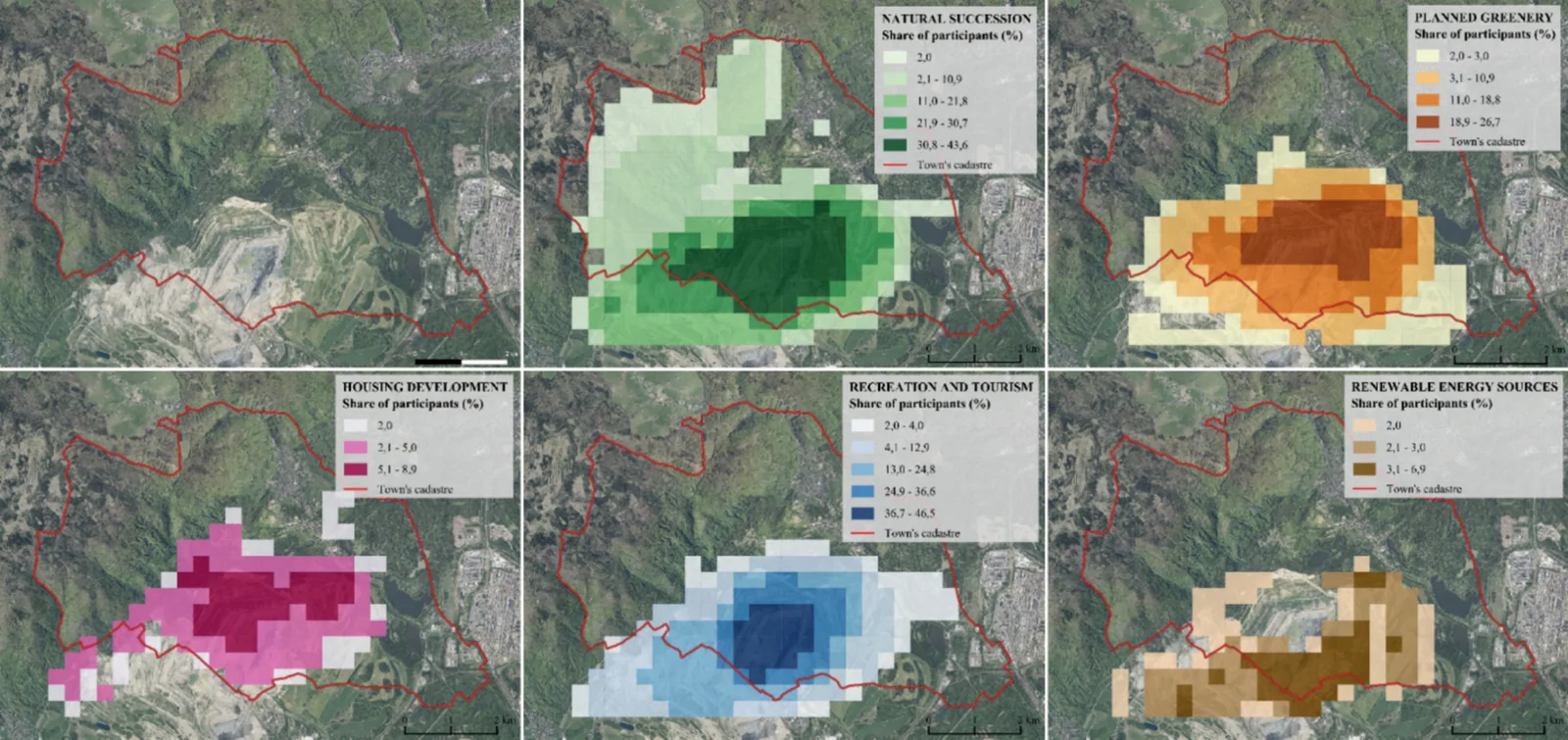
Spatial distribution and intensity of five post-mining land-use scenarios. *Note*: The first map provides a baseline aerial view and cadastre boundaries (red line) for spatial context. *Source*: authors’ survey and elaboration

For the thematic maps showing the spatial distribution and intensity of each land-use category (Fig. 5), a minimum threshold of two participants per grid cell was applied to enhance analytical accuracy and ensure patterns reflect minimal consensus rather than isolated preferences. This approach reveals not only where each land-use type was preferred but also the relative strength of these preferences as indicated by participant percentages for specific locations. This methodology enables analysis of preference overlaps and the relative popularity of each land-use category.

**Fig. 6:**
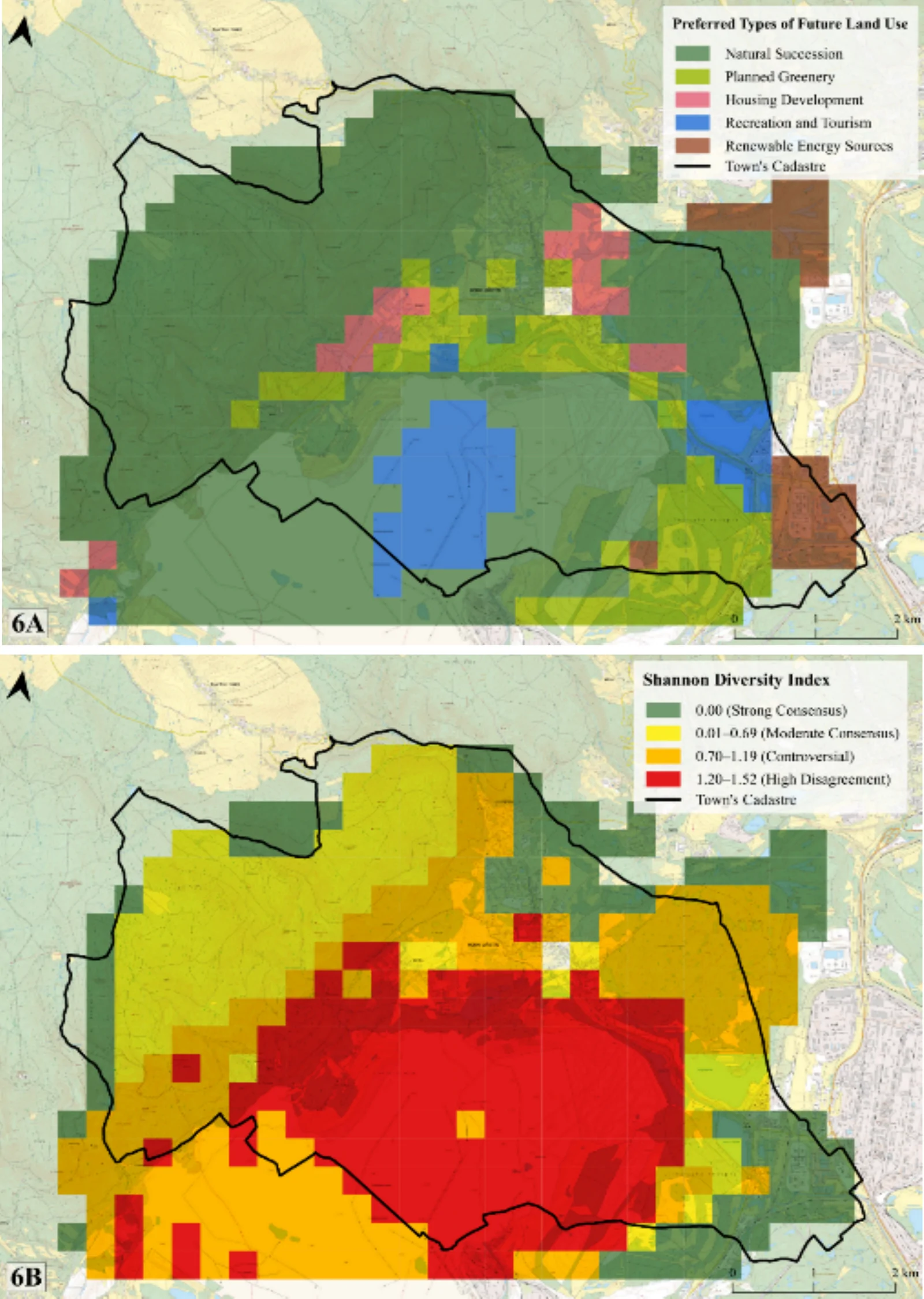
Dominant post-mining land-use preferences (**A**) and participant agreement levels (**B**) based on Shannon Diversity Index analysis. *Source*: authors’ survey and elaboration

In contrast, the synthesis analysis (Fig. 6) employed a majority-rule aggregation approach, in which each grid cell displays the most frequently selected land-use preference among participants. For instance, if 15 participants marked a particular cell with 5 preferring natural succession, 3 selecting planned greenery, and 7 choosing recreation and tourism, the cell was assigned to the recreation and tourism category.

To quantify spatial patterns of agreement and disagreement, the Shannon Diversity Index was applied to measure participant agreement levels within each grid cell, calculated as *H* = − Σ(pi × ln(pi)), where pi denotes the proportion of participants selecting each land-use category. Values range from 0 (perfect consensus) to 1.52 (maximum disagreement), with the resulting values classified into four categories. Controversy categories were established using natural breaks (Jenks optimisation) of the Shannon diversity index values calculated for all grid cells containing participant responses (*n* = 421). This approach ensures objective classification based on the actual distribution of diversity values in our study area, in accordance with established practices in participatory mapping research (Fagerholm et al. 2012).

## Results

### Spatial distribution and intensity of land use preferences

Natural succession emerged as the most strongly supported land-use option, with 30.8–43.6% of participants (dark green) designating the central quarry area for this purpose. This concentrated support indicates substantial community preference for allowing ecological processes to guide landscape recovery in the most heavily disturbed mining zones.

Recreation and tourism preferences displayed similarly high consensus levels, with 36.7–46.5% of participants (dark blue) selecting the central quarry area for this purpose. This spatial overlap with natural succession preferences suggests participants envision nature-based recreational opportunities rather than intensive tourism development. Additionally, the concentric pattern of decreasing support moving outward from the quarry centre indicates a clear spatial focus for recreational development.

Planned greenery received substantial support (a maximum of 18.9–26.7%) in a different spatial pattern that complements rather than overlaps with natural succession areas. Participants favoured designed green spaces primarily in the northern portion of the post-mining area, creating a potential buffer zone between more naturalised areas and the existing town’s settlements.

Housing development preferences were spatially concentrated in the northern and northwestern sections of the post-mining area, with low support levels (maximum of 5.1–8.9%). Interestingly, these areas are not directly adjacent to existing built-up zones of the town, suggesting participants envision discrete new residential nodes rather than contiguous urban expansion. The partial overlap with planned greenery preferences suggests a desire for integrated housing–greenspace development with enhanced environmental quality.

Renewable energy development and related industrial production received the lowest overall support, with maximum consensus reaching only 3.1–6.9% in scattered locations (dark brown). This limited and dispersed pattern indicates minimal community interest in industrial or energy development within the post-mining landscape, reinforcing the predominant preference for nature-oriented restoration approaches.

### Dominant land use preferences and consensus pattern: A synthesis

The resulting synthetic map (Fig. 6A) reveals distinct spatial patterns in community preferences, with natural succession emerging as the overwhelmingly preferred strategy across 297 cells (70.5% of the analysed cells). This preference encompasses large portions of the post-mining landscape and the forested regions in the northern, northwestern, and eastern sections of the cadastre. Recreation and tourism preferences (35 cells, 8.3%) dominated the central post-mining area, reflecting continued support for the original quarry-flooding restoration plan. In contrast, planned greenery preferences (50 cells, 11.9%) formed clusters along the quarry perimeter and in the southern intravillane zone. Housing development and renewable energy installations received comparatively limited support, with housing preferences (17 cells, 4.0%) concentrated within existing urbanised areas and renewable energy (22 cells, 5.2%) relegated to the eastern periphery.

However, the majority-rule approach masks important variations in participant agreement. To reveal the actual level of consensus behind these dominant preferences, we calculated the Shannon Diversity Index for each grid cell (Fig. 6B). This analysis categorised cells into four agreement levels, ranging from strong consensus (0.00) to high disagreement (max. 1.52). The highest disagreement concentrates in the central quarry area and its immediate surroundings, with the rest of the quarry falling within the second-highest disagreement category. Notably, no moderate- or strong-consensus cells appeared within the quarry area itself, indicating substantial variability in residents’ visions for post-mining spaces. This may reflect varying levels of participant familiarity with restoration plans, as some residents were well informed about current proposals, whereas others appeared unaware of the planning documents. The level of participant agreement increases with distance from the quarry. Strong- and moderate-consensus cells predominate in the forested northwestern areas. Conversely, the intravillane areas exhibit controversial-to-high disagreement values.

This spatial distribution indicates that, although natural succession appears dominant in the synthetic map, the post-mining core remains highly contested. Quantitatively, the analysis reveals substantial spatial variation, with 47.0% of cells showing consensus (17.6% perfect, 29.5% moderate), whereas 53.0% exhibit disagreement (20.4% controversial, 32.5% high disagreement). The Shannon Diversity Index analysis demonstrates the methodological value of measuring not only what communities prefer, but also where they agree. Such spatial analysis of consensus enables planners to strategically target stakeholder engagement in controversial locations while proceeding with implementation in areas of agreement.

### Differences in land use preferences between sociodemographic groups

**Fig. 7:**
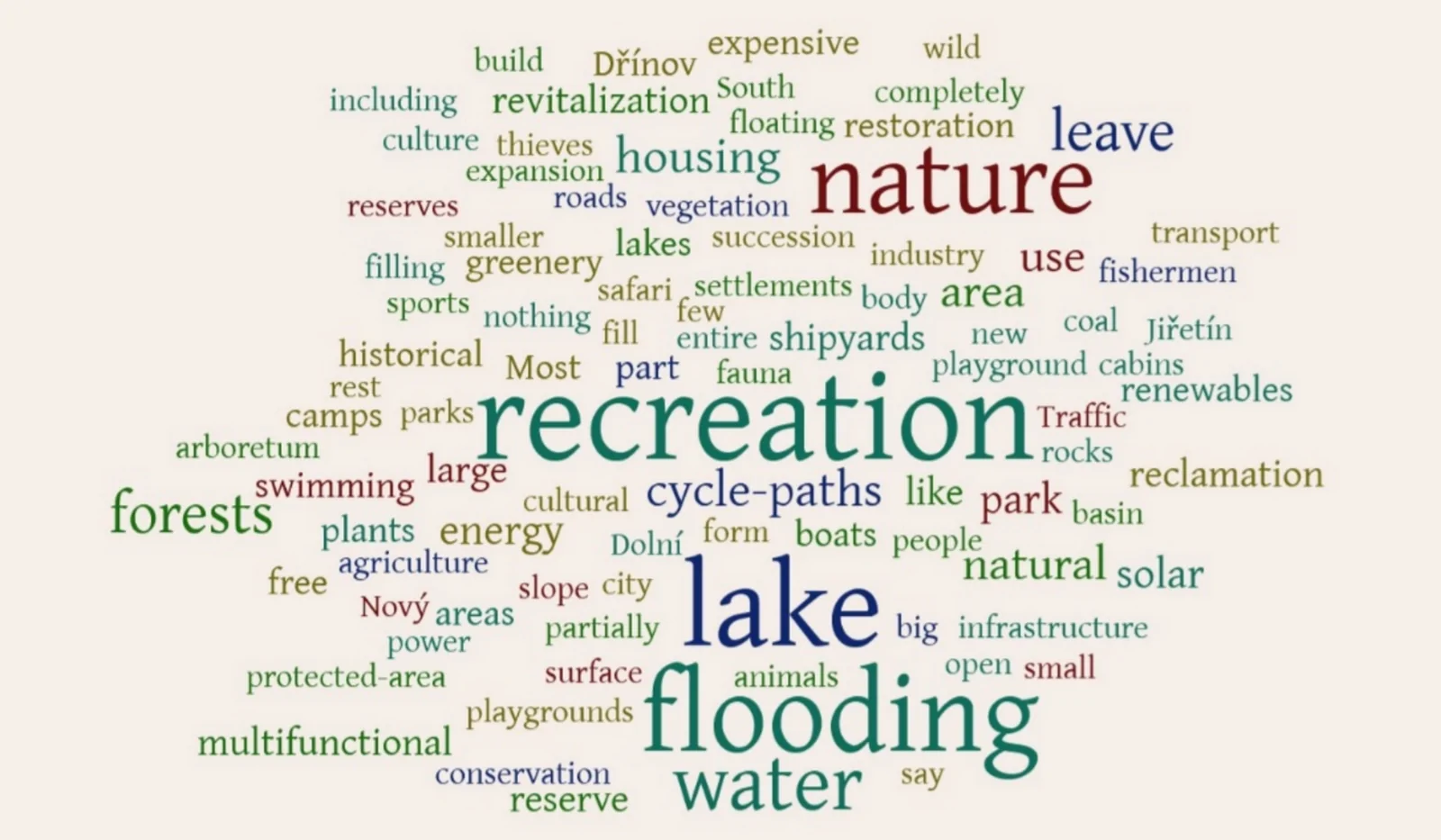
Wordcloud of preferences for the future use of post-mining landscape. *Source*: authors’ survey and elaboration

When we asked respondents a completely open question (with no answer options) about what purpose the area should be used for after coal mining in their opinion, most respondents (about 70%) mentioned some form of flooding, the creation of a lake or generally some water area (see Fig. 7). This has been the most common way of open-cast mining landscape reclamation in the Czech Republic, but also in neighbouring Germany, in recent decades (Frantál et al. 2024). Participant responses varied regarding the scale of flooding, ranging from a preference *“to flood all the mine with water”* to a desire to *“leave only a smaller body of water and start new housing development around it”.* Furthermore, visions differed concerning the lake’s filling process, whether through natural precipitation or artificial irrigation, and its intended functions, such as recreation, fish farming, or floating solar power plants.

Most people would prefer multifunctional recreational use of the landscape, including the creation of a lake with surrounding forests and green areas, along with cycle paths and tourist and sports facilities (e.g. cabins, campsites, playgrounds, shipyards). Another significant difference in preferences concerned the degree of human intervention in the landscape and the extent of the area that should be (according to the plans of the Ministry of the Environment) left to natural succession and the creation of a natural reserve. Approximately 15% of respondents explicitly stated that the entire area should be left to nature (some added that nature would manage it best). On the other hand, a similar proportion of respondents emphasized the need for new housing construction and renovation of road infrastructure in the area. Several respondents mentioned the potential for developing renewable energy sources, but a few explicitly stated that they do not prefer floating (on the lake) solar power plants.

In the following question, respondents rated the five predetermined categories on a scale according to their preferences (see Table 1). Notably, leaving the post-mining area to nature (for natural succession) received the highest average priority ($\overline{x}$ = 3.92) among respondents. The second most preferred type of land use ($\overline{x}$ = 3.79) was planned greenery, encompassing various forms of parks, forest parks, and similar areas.

**Table 1:** Differences in the preference for specific post-mining land uses between groups according to age, education, and length of stay in the area. ^a^Result of the ANOVA: mean values (for categories of age, education, and length of stay), F-statistic, significance, and Eta correlation coefficient. Statistically significant differences in mean values across specific categories are shown in bold. The degree of preference was measured on a scale from 1 (very low priority) to 5 (very high priority). *Source*: Authors’ survey and calculations

Post-mining land use	Mean (total)	Variance	Category	Groups	ANOVA^a^			
					Mean	F	Sig	Eta
Natural succession	3.92	1.59	Age	< 30 years	3.81	0.170	0.844	0.059
				30–49 years	3.85			
				50 + years	3.98			
			Education	Elementary	4.15	0.315	0.731	0.081
				Secondary	3.86			
				Tertiary	4.00			
			Length of residence	Long-term	3.75	0.853	0.358	0.093
				Short-term	4.00			
Planned greenery	3.79	1.11	Age	< 30 years	3.56	0.723	0.488	0.122
				30–49 years	3.96			
				50 + years	3.77			
			Education	Elementary	**3.08**	**4.125**	**0.019**	**0.281**
				Secondary	**3.85**			
				Tertiary	**4.17**			
			Length of residence	Long-term	3.97	1.402	0.239	0.119
				Short-term	3.70			
Recreation and tourism	3.32	1.77	Age	< 30 years	2.81	2.022	0.138	0.201
				30–49 years	3.65			
				50 + years	3.32			
			Education	Elementary	**3.38**	**2.413**	**0.045**	**0.219**
				Secondary	**3.19**			
				Tertiary	**4.08**			
			Length of residence	Long-term	3.16	0.742	0.391	0.087
				Short-term	3.40			
Renewable energy production	2.35	1.66	Age	< 30 years	2.31	0.042	0.959	0.030
				30–49 years	2.31			
				50 + years	2.39			
			Education	Elementary	1.92	1.096	0.338	0.149
				Secondary	2.38			
				Tertiary	2.67			
			Length of residence	Long-term	2.16	1.110	0.295	0.106
				Short-term	2.45			
Housing development	2.29	2.03	Age	< 30 years	**3.19**	**4.380**	**0.015**	**0.289**
				30–49 years	**2.31**			
				50 + years	**2.04**			
			Education	Elementary	2.54	0.443	0.643	0.096
				Secondary	2.30			
				Tertiary	2.00			
			Length of residence	Long-term	**1.78**	**6.452**	**0.013**	**0.250**
				Short-term	**2.54**			

The third most preferred variant ($\overline{x}$ = 3.32) was the use of the landscape for recreation and tourism development. Variants of using the landscape for new energy production (renewable energy sources) and for new construction and housing development received significantly lower priority (on average, around 2.3). While there was relatively high agreement among respondents on support for planned green spaces, the highest degree of variance (2.03) was observed in responses regarding potential new housing development.

To some extent, there were differences in preferences for specific types of land use depending on age, education, and the length of living in the place of residence (see Table 1). While there were no significant differences between sociodemographic groups in relation to natural succession, the preference for planned greenery increases significantly with the level of education attained. Similarly, the preference for recreation and tourism is statistically significantly higher among people with a university education than among those in other educational categories. Mean values of the support for renewable energy production also increase with the level of education; however, these differences did not prove to be statistically significant. The housing development is more likely preferred by short-term residents compared to old settlers and also by younger people (up to 30 years old). Minor differences in preferences by gender and socioeconomic status are not statistically significant.

## Discussion

This research aimed to identify spatial patterns in residents’ preferences across various post-mining land-use scenarios. Specifically, we examined the level of support for natural succession versus managed reclamation and whether these preferences differ by socio-demographic characteristics. Residents’ opinions (as reflected in spatial preferences in mental maps and in complementary information obtained from questionnaires and interviews) reflect the post-mining land-use dilemmas that experts also face. The two sources of disputes concern (i) the extent and process of flooding of the area, including the future use of the lake, and (ii) the extent of the area left to natural succession, the degree of human intervention, and the use of the future nature reserve. While there was strong consensus on the general preference for flooding the quarry to create a lake, this vision held different meanings for participants. For some, the lake was a natural reserve surrounded by forest, whereas for others it was associated with intensive recreational use, including shipyards, cottages, campsites, and renewable energy installations.

In Central Europe, former quarries have usually been flooded to create more or less extended artificial lakes that can serve various functions and ecosystem services (Pešout et al. 2022; Frantál et al. 2024). When emphasizing the creation of the lake, respondents mentioned (during interviews) examples of already existing post-mining lakes in the region (e.g. lakes Most and Matylda in the near city of Most), which are popular for summer recreation. While post-mining lakes can be spectacular examples of renewal (Deshaies 2018), in different regional contexts and local conditions, such lakes may also suffer from limited water resources or poor water quality, and some projects have proven environmentally and economically unsustainable (Frantál et al. 2024).

The problem of lake formation or rather the process of its flooding (whether by slow filling from natural sources or by technical means via artificially created channels) is closely related to the general dilemma of technical reclamation versus natural succession. Disputes over the merits of technical reclamation and natural succession often hinge on differing interpretations of the processes and outputs of reclamation projects (Sádlo and Gremlica 2017; Harries et al. 2024). Among the main benefits of technical reclamation emphasized is the speed of landscape recovery for new human use. Critics argue that extremely expensive reclamation measures, based on extensive technical modifications to the terrain, destroy the natural components of the landscape, impoverish species diversity and habitat diversity, and undermine the overall structure of landscapes. Proponents of reclamation counter that the “technical” is merely a pejorative label and that reclamation has always been an interplay of technology and ecology, with a preference for greenery and lakes as the main elements of the landscape (Štýs 2009). On the other hand, the critics of spontaneous succession and the creation of protected natural reserve (as a “new wilderness”) on a significant part of the area emphasize the issue of preventing access to the landscape and its use by local communities.

Our research has shown that support among local residents for leaving part of the post-mining area to natural succession is relatively high, in contrast to the proclamations of some regional civic associations and protest groups (see Klusáček et al. 2025). The key question is to what extent this succession should be carried out? Our results align with recent studies from Poland (Krzysztofik et al. 2020; Kantor-Pietraga et al. 2023), which highlight the community preference for multifunctional recycling and the “polarisation of functions”. This approach involves designating specific land and water bodies for recreational or economic use while preserving other areas for biodiversity and natural values, where only low-impact activities such as walking and angling are permitted (Kantor-Pietraga et al. 2023).

Even within our relatively small sample, there were statistically significant differences in the level of support for certain types of land use between sociodemographic groups. Specifically, the housing development is more strongly supported by young people under thirty and by those living in the locality for a shorter period. We hypothesize that these individuals are currently considering purchasing properties and are more aware of the need for new housing construction. This appears to be consistent with broader trends in the housing market (e.g. Opit et al. 2020). Support for the planned greenery (parks, forest parks), recreational use, and tourism increases significantly with higher levels of education. Somewhat surprisingly, there were no significant differences between educational categories in support for natural succession and renewable energy development. Some studies have reported that the most important sociodemographic factor influencing perceptions and evaluations of the physical attributes of landscapes is people’s professional field or study focus (e.g. Svobodová et al. 2012). In this regard, it would be appropriate to conduct future research using larger samples and to account for respondents’ education background and profession in the analysis.

### Methodological limitations of the approach

Several limitations of this study should be acknowledged. First, participatory mapping approaches are susceptible to map literacy challenges, in which participants may experience difficulty navigating printed maps that lack the search and zoom functionalities available in some digital or in-situ participatory tools (Brisudová et al. 2024). While we attempted to minimise this risk by assisting during mapping activities, complete elimination of this limitation was not possible. This may have affected the spatial precision of some participants’ responses, particularly among those less familiar with map-based representations. To address literacy and skill disparities, Svobodová and Everingham (2026) recommend implementing a “capacity-building”, including training sessions or facilitated workshops where community members can co-create spatial data with guidance. A mixed-methods approach blending PPGIS with interviews (as in the case of our study) or other qualitative methods, such as focus groups, photovoice, and ethnographic insight, can help to apply opinions also to those less comfortable with maps and technologies (*cf.* Svobodová and Everingham 2026).

Second, social desirability bias represents a potential concern, as participants may provide responses they perceive as socially acceptable rather than expressing their genuine preferences (Solymosi et al. 2022). This bias could be particularly relevant in post-mining contexts where environmental restoration and natural succession are generally viewed favourably, potentially leading to underreporting of preferences for other development-oriented land uses. Although some research suggests gender-related variations in social desirability bias (Sutton and Farrall 2005), our study design did not permit examination of this phenomenon. Future research should investigate the influence of social desirability and other potential biases on community attitudes towards post-mining restoration better to understand the complexity of preference formation in these contexts.

Third, the prolonged planning history of the Horní Jiřetín coal mining area may have influenced participants’ responses. Over several decades, various restoration scenarios have been presented to stakeholders and the public, with proposals varying according to available financial resources and changing policy priorities (Klusáček et al. 2025). For example, the most recent official restoration plan proposed post-mining lakes in the former quarry area, a scenario that received strong support in participants’ mapping outcomes. This alignment suggests that participants’ prior exposure to official restoration plans, regardless of their current validity or feasibility, may have anchored their preferences towards previously proposed options rather than encouraging entirely independent visioning. This potential anchoring effect could have constrained the diversity of community-generated alternatives, as participants may have consciously or unconsciously regarded officially presented scenarios as more legitimate or feasible than other approaches. Consequently, our findings may reflect both genuine community preferences and expectations shaped by previously presented planning proposals, potentially limiting the emergence of truly novel restoration ideas.

## Conclusion and policy implications

Post-mining regions globally face the dual challenges of ecological restoration and socioeconomic transition (Wirth et al. 2012; Alves Dias et al. 2018; Li et al. 2025). In this context, participatory mapping approaches, as presented in this study, offer valuable tools for ensuring that regeneration efforts reflect not only technical, ecological, and economic imperatives but also honour the lived experiences, values, and aspirations of affected communities, ultimately leading to more sustainable and socially acceptable outcomes.

In terms of spatial extent, the natural succession and recreation/tourism categories covered the largest areas, dominating the central and peripheral portions of the post-mining landscape. Planned greenery occupied a moderate spatial coverage, whereas housing development and renewable energy installations were the most spatially constrained, reflecting limited support among participants. The level of consensus also followed this pattern, with natural succession and recreation achieving the highest agreement levels (up to 46%), planned greenery showing moderate consensus (up to 27%), and housing and renewable energy options demonstrating notably lower agreement levels (< 10%), indicating stronger community alignment around nature-based and recreational restoration approaches.

This study yields several policy implications relevant for post-mining land-use planning that could apply to similar coal-dependent regions undergoing just transition processes. First, participatory mapping approaches can promote more equitable energy transition governance by including communities in decision-making processes and recognizing their local expertise. Our study demonstrates that residents of Horní Jiřetín town possess detailed spatial knowledge about their post-mining landscape. They expressed nuanced preferences that distinguish between areas suitable for natural succession versus designed green spaces while carefully constraining development to specific zones. This local knowledge is often missing from top-down planning processes. Including residents in post-mining land-use planning reduces feelings of exclusion from decisions affecting their communities, which is often a critical concern in former coal mining regions that have historically served as energy sacrifice zones. A similar collaborative approach can enhance the social acceptance of restoration decisions, even when outcomes differ from community preferences, as residents perceive the process as democratically legitimate.

Second, our findings reveal significant knowledge gaps among community members regarding official restoration plans, with some participants being well informed about current proposals, while others referenced outdated information. This uneven access to information undermines effective participation and can cause social inequities in energy transition processes. Transparent and regularly updated communication about post-mining restoration plans is therefore essential for meaningful community engagement. Energy transition policies should prioritise clear information sharing to enable genuine participation in post-coal landscape planning.

Identifying the optimal scale of landscape alteration is necessary to exploit its potential for regional rebranding and economic revitalisation, while simultaneously safeguarding intrinsic identity elements and adhering to environmental justice standards. In this context, it is important to evaluate the planned regeneration projects that apply for financing from the Just Transition Funds responsibly and comprehensively and to ensure that supported redevelopment activities do not reproduce old disparities and injustices or create new ones (see Bird and Cassier 2023).

The objective of our study was primarily to test the given methodological approach, its advantages and limitations. However, in future practical uses it is very important to deal with the issues of influence and practical accountability. Participatory mapping raises people’s expectations that expressed values and opinions will shape outcomes, and if local community-generated maps are ignored or deprioritised in the decision-making, it can deepen mistrust and deprivation (*cf.* Svobodová and Everingham 2026).

This one of the first empirical studies applying participatory mapping in the context of post-mining landscape restoration may represent the basis for an empowering vision of the just transition from below. This means not only stating that no person or place should be left behind, but also devising ways to recognise how people are linked to places and landscapes, how these links change, and what this means for the affected communities. Raising the visibility and legitimacy of local voices in the just transition is a crucial step towards moving beyond the reproduction of sacrifice zones and towards a more democratic and just vision of post-coal development.
